# Assessment of the Efficacy and Safety of CyberKnife Radiosurgery on Optic Nerve Sheath Meningiomas

**DOI:** 10.3390/vision10040080

**Published:** 2026-10-11

**Authors:** Yuhang Wang, Xintong Xu, Shanshan Cao, Mingming Sun, Chunyan Pan, Quangang Xu, Shihui Wei, Zhiqing Li, Longsheng Pan, Huanfen Zhou

**Affiliations:** 1 Senior Department of Ophthalmology, Chinese PLA General Hospital & Chinese PLA Medical School, Beijing 100853, China; 2 Tianjin Key Laboratory of Retinal Functions and Diseases, Tianjin Branch of National Clinical Research Center for Ocular Disease, Eye Institute and School of Optometry, Tianjin Medical University Eye Hospital, Tianjin 300384, China; 3 Department of Ophthalmology, Beijing Anzhen Hospital, Capital Medical University, Beijing 100029, China; 4 Department of Ophthalmology, Air Force Medical Center, Beijing 100142, China; 5 Senior Department of Neurosurgery, Chinese PLA General Hospital & Chinese PLA Medical School, Beijing 100853, China

**Keywords:** optic nerve sheath meningioma, CyberKnife radiosurgery, fractionated stereotactic radiotherapy, visual outcomes, neuro-ophthalmology

## Abstract

The purpose of this study was to evaluate the efficacy and safety of CyberKnife (CK) radiosurgery in patients with optic nerve sheath meningiomas (ONSM). We retrospectively identified patients with ONSM treated with CK between January 2020 and January 2024 from the Database of Chinese Neuro-Ophthalmic Diseases. CK was delivered at 23–25 Gy in 5 fractions. Best-corrected visual acuity (BCVA) and visual field mean deviation (VF MD) were assessed before treatment and at the final follow-up, with a median visual follow-up of 36 months (range, 26–64 months). Tumor volume and CK-related complications were also evaluated. Twenty patients (22 affected eyes; 2 with bilateral involvement) were included. Treatment success was achieved in 90.9% (20/22) of eyes, including improvement in 59.1% (13/22) and stable visual function in 31.8% (7/22), whereas 9.1% (2/22) worsened. Among the 12 eyes with available VF MD data, 91.7% (11/12) improved and 8.3% (1/12) worsened. Spearman correlation analysis revealed that poorer baseline BCVA (*r* = 0.457, *p* = 0.032) and a longer interval from onset to CK (*r* = 0.450, *p* = 0.036) were associated with poorer visual outcomes, whereas age and tumor volume were not significantly associated with visual changes (*p* > 0.05). MRI follow-up was available for 13 patients (14 eyes), with tumor control achieved in all 14 eyes. No serious treatment-related complications were observed. CK hypofractionated radiosurgery may preserve and improve visual function in ONSM, providing a potentially safe and effective treatment option.

## 1. Introduction

Optic nerve sheath meningiomas (ONSM) are rare benign tumors that originate from the cap cells of the arachnoid that surround the optic nerve. About 95% of ONSMs are unilateral. Although they are slow-growing, their location can directly affect the anterior optic nerve and lead to progressive and severe visual loss [1].

Patients may be observed without treatment if the visual loss is not progressing or if the eye is blind and the tumor is confined to the orbit [2,3]. Attempted surgical extirpation of the meningioma carries a high risk of blindness [4]. However, surgery may be considered in selected cases, such as those with disfiguring proptosis or significant intracranial extension [5]. Recently, transnasal endoscopic optic nerve decompression has been proposed and has shown stabilization of disease and, in some cases, improvement in visual function compared with baseline [6,7].

Standard-fractionated radiotherapy has shown its efficacy in controlling ONSM tumor growth while allowing visual acuity to improve or stabilize. However, radiation-induced toxicity may ultimately jeopardize the functional benefit [8]. Recently, stereotactic radiotherapy (STR) has been employed to deliver the appropriate amount of radiation to the tumor in a localized fashion. This approach is effective and non-invasive and reduces exposure to surrounding tissue by enabling modulation of dose intensity within the radiation field. Several studies have shown that visual function improved or was preserved after STR, including intensity-modulated radiation therapy (IMRT) [9], conventional fractionated radiotherapy with Novalis or hypofractionated radiotherapy with CyberKnife (CK) [10]. CyberKnife provides image-guided, highly conformal stereotactic irradiation, which may reduce radiation exposure to adjacent ocular and orbital structures. However, clinical evidence regarding hypofractionated CK treatment for ONSM, particularly long-term visual outcomes, remains limited [11]. Therefore, the present study examined the efficacy and complications associated with CK for ONSM by applying the technique and comparing visual function before and after treatment.

## 2. Materials and Methods

### 2.1. Patient Selection

We used the Database of Chinese Neuro-Ophthalmic Diseases (Registration Number: 00376-2020) to collect clinical information from patients diagnosed with ONSM between January 2020 and January 2024 from the People’s Liberation Army General Hospital (PLAGH) in China.

The diagnosis of ONSM was based on characteristic clinical manifestations and orbital MRI findings. Exclusion criteria included one or more of the following: history of tumor biopsy or surgical resection; prior radiation therapy for tumors; refusal of CK treatment; presence of other ocular diseases, including significant cataract, that could affect visual acuity (VA) or visual field (VF).

We analyzed the clinical characteristics and examinations of patients with ONSM before and after CK radiotherapy. Informed consent was obtained from all patients. The study protocol was approved by the institutional review board of Chinese PLA General Hospital (S2021-127-01) and was performed in accordance with the guidelines contained in the Helsinki Declaration.

### 2.2. Ophthalmological and Associated Examinations

Best-corrected visual acuity (BCVA) was assessed in all patients using a Snellen chart and converted to logarithm of the minimum angle of resolution (logMAR) values. Visual acuities of counting fingers (CF), hand motion (HM), light perception (LP), and no light perception (NLP) were recorded as 2.6, 2.7, 2.8, and 2.9 logMAR, respectively. Visual field (VF) assessment was performed using a Humphrey Field Analyzer 3 (Carl Zeiss Meditec, Dublin, CA, USA) with the Central 24-2 SITA Faster strategy using a Goldmann size III white stimulus, covering the central 24° of the visual field with nasal test points extending to 30°, and visual field mean deviation (VF MD) was used as the quantitative VF parameter. VF tests were considered reliable when fixation losses were <20% and false-positive and false-negative rates were < 33%. Only eyes with reliable VF examinations both before and after CK treatment were included in the VF MD analysis, resulting in 12 eligible eyes. Peripapillary retinal nerve fiber layer (RNFL) thickness was assessed using spectral-domain optical coherence tomography (Cirrus HD-OCT, Carl Zeiss Meditec, Dublin, CA, USA) with the Optic Disc Cube 200 × 200 protocol.

### 2.3. Optic Nerve MRI

All patients underwent orbital magnetic resonance imaging (MRI) using a 3.0-T scanner (Discovery 750, GE Healthcare, Milwaukee, WI, USA). The imaging protocol included fat-suppressed T2-weighted imaging (T2WI), fat-suppressed T1-weighted imaging (T1WI), and gadolinium-enhanced T1-weighted imaging. MRI is the principal imaging modality for the diagnosis of ONSM. In patients with characteristic clinical and radiological findings, a presumptive diagnosis can often be established without tissue biopsy. On MRI axial images, ONSM typically presents with the characteristic “tram-track” sign, which corresponds to the enhancing outer ONSM encircling the inner non-enhancing optic nerve. On the coronal images, this appears as a “doughnut” or “non-enhancing dot” sign [12].

### 2.4. Treatment

All patients received CK radiosurgery. Prior to treatment, MRI was performed for target localization, followed by immobilization mold fabrication and positioning using a dedicated computed tomography (CT) simulator (Siemens Healthineers, Forchheim, Germany). A stereotactic radiosurgery plan was jointly developed by neurosurgeons and radiologists, with the planning target volume (PTV) encompassing the ONSM. Before treatment, patients and their family members were fully informed of the disease condition, radiotherapy-related precautions, and potential adverse effects, and written informed consent was obtained. CT images were acquired with a slice thickness of 1.0 mm. The gross tumor volume (GTV) was delineated based on tumor extent identified on diagnostic MRI and/or CT imaging. A margin of 0–3 mm was added to the GTV to generate the PTV. The prescribed radiation dose ranged from 23 to 25 Gy and was generally delivered in five fractions over five consecutive days. Tumor volume was measured using the treatment planning system before and after treatment, and volumetric changes were assessed whenever follow-up imaging was available.

The median total CK dose was 23 Gy (range: 23–25 Gy), with a median of 5 fractions.

### 2.5. Data Collection

The following data were collected from electronic medical records: patient gender, age at symptom onset, laterality, side of the affected eye, interval from symptom onset to presentation, time interval from symptom onset to CK initiation, radiotherapy dose and number of sessions, time interval from CK initiation to follow-up (months), and physical signs and symptoms at the time of presentation.

### 2.6. Statistical Analysis

The results were analyzed using IBM SPSS Statistics Software version 27.0 (IBM, Armonk, NY, USA). For normally distributed data, continuous variables were summarized as mean ± standard deviation (SD); for non-normally distributed data, they were summarized as median (range). Analyses were performed using paired *t*-tests or signed-rank tests based on the distribution of the data. Spearman correlation analysis was used to analyze baseline clinical factors associated with visual acuity change (∆VA). A *p*-value < 0.05 was considered statistically significant.

## 3. Results

### 3.1. Clinical Features

A total of 20 patients were enrolled in the study, with a higher proportion of female patients, including 15 females (75.0%) and 5 males (25.0%). The mean age at onset was 40.2 ± 15.6 years (range: 9–79 years). Unilateral ONSM was present in 18 patients (90.0%), while bilateral involvement was observed in 2 patients (10.0%). Accordingly, a total of 22 affected eyes were analyzed, with 18 eyes (81.8%) affected unilaterally and 4 eyes (18.2%) bilaterally. The median interval from symptom onset to presentation was 13.5 months (range: 2–197 months), and the median interval from symptom onset to initiation of CK treatment was 15 months (range: 2–198 months). The clinical characteristics of the patients are summarized in Table 1.

**Figure 1:**
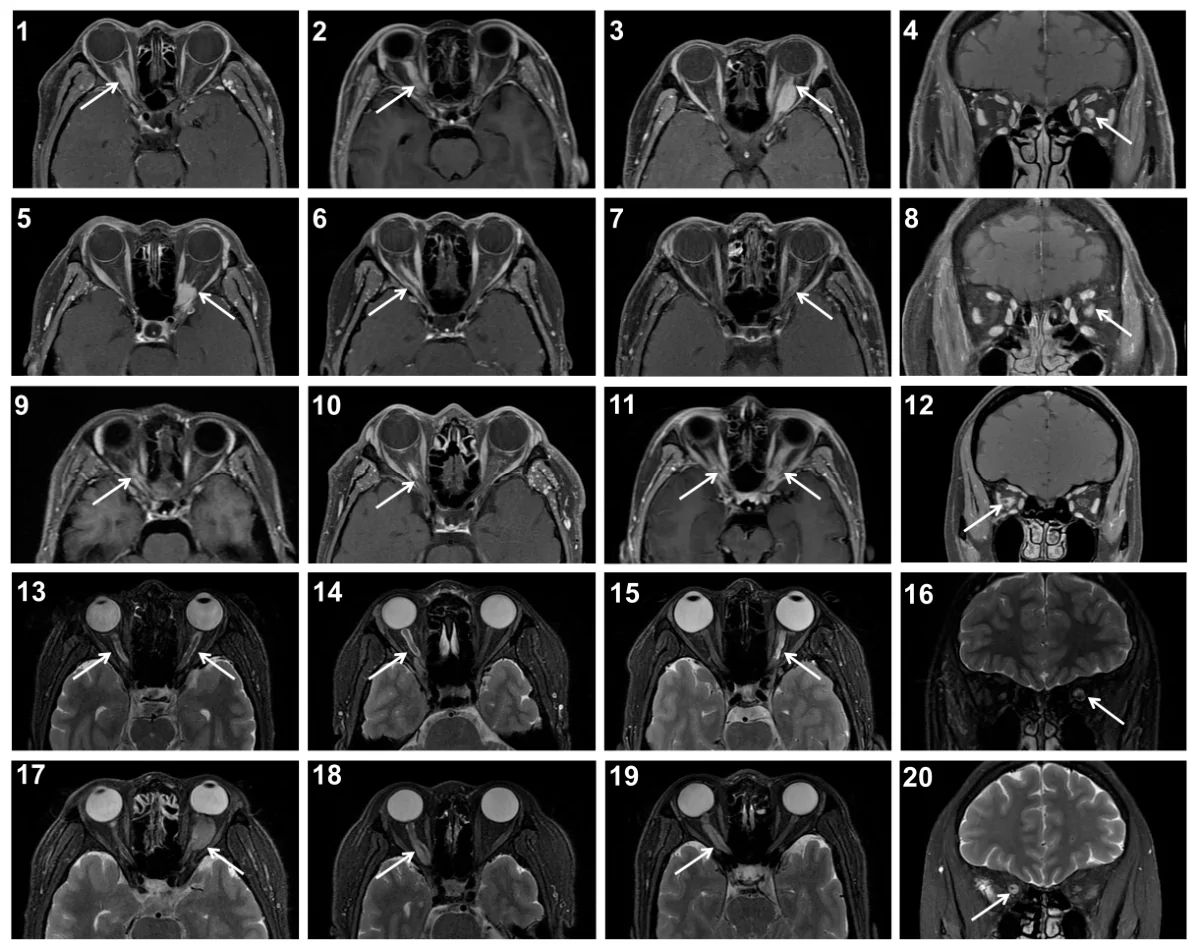
MRI findings demonstrating typical imaging features of ONSM in patients 1–20. Post-contrast T1-weighted imaging (axial planes 1, 2, 3, 5, 6, 7, 9, 10, 11 and coronal planes 4, 8, 12) demonstrates unilateral or bilateral enhancement of the optic nerve (arrows). Axial fat-suppressed T2-weighted images (axial planes 13, 14, 15, 17, 18, 19 and coronal planes 16, 20) reveal increased signal intensity within the optic nerves (arrows). Patients 11 and 13 present with bilateral optic nerve sheath meningiomas.

The primary symptoms presenting at the initial visit included decreased visual acuity (90.9%) and visual field defects (59.1%). The optic disc abnormalities at the first visit included optic disc edema (15/22 eyes, 68.2%) and optic disc pallor (6/22 eyes, 27.3%). Only one affected eye (4.5%) showed no significant optic disc abnormalities. Additionally, one eye (4.5%) exhibited exophthalmos. Typical imaging features of ONSM are shown in Figure 1.

### 3.2. Visual Function

**Figure 2:**
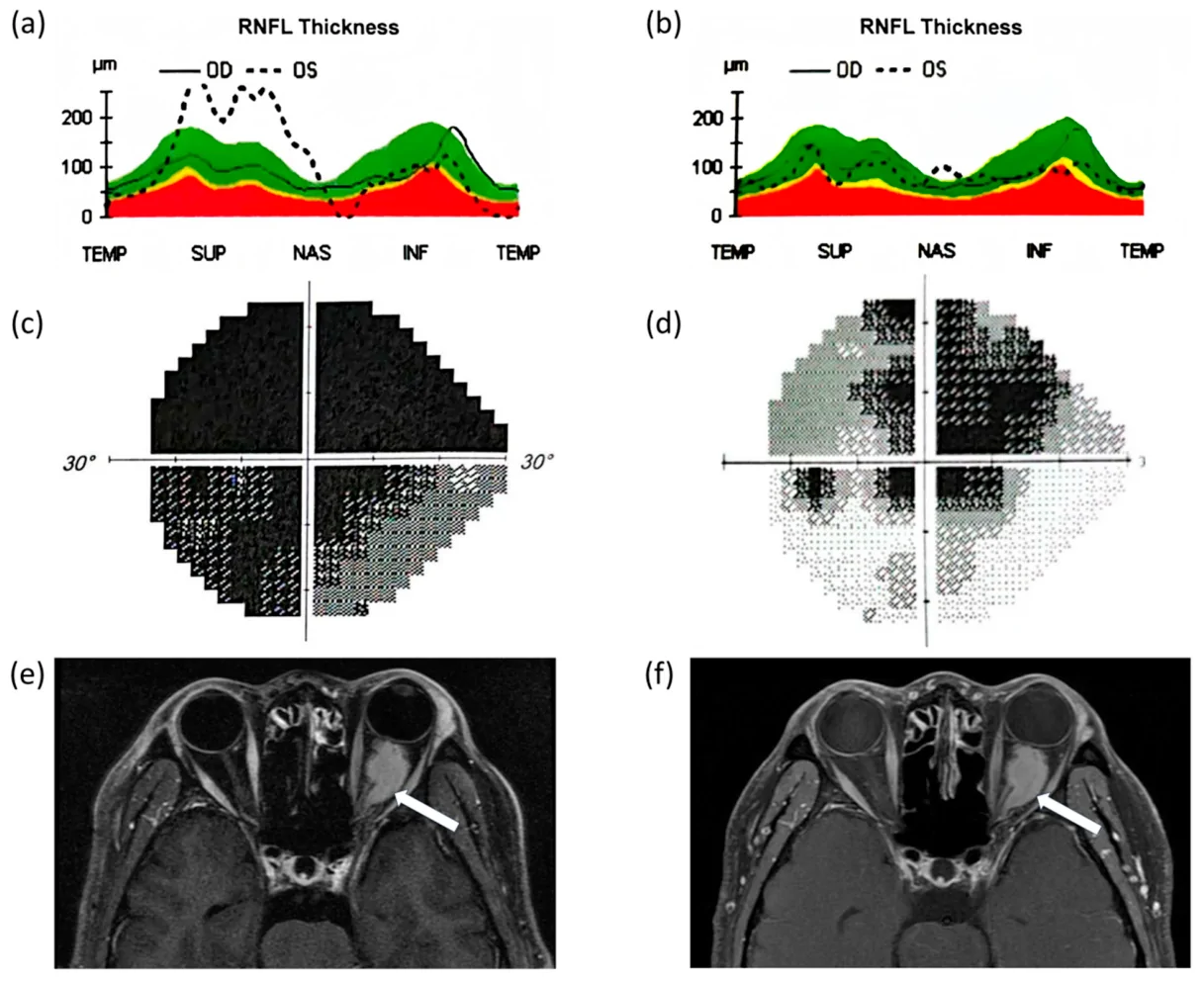
Representative ophthalmic and neuroimaging findings of Patient 17 before and after CK treatment. (**a**,**b**) Peripapillary RNFL thickness on optical coherence tomography before and after CK treatment, respectively. (**c**,**d**) Visual field examinations before and after CK treatment, respectively. (**e**,**f**) Axial contrast-enhanced T1-weighted orbital MRI before and after CK treatment, respectively. White arrows indicate the enhancing optic nerve sheath meningioma. At baseline, BCVA was 0.6 logMAR and VF MD was −29.72 dB. At the 2-year follow-up after CK treatment (23 Gy in 5 fractions), BCVA improved to 0.3 logMAR and VF MD improved to −19.78 dB. Follow-up MRI showed a slight reduction in tumor size and decreased enhancement of the optic nerve sheath.

Baseline visual function was characterized by a median pre-CK BCVA and a mean pre-CK VF MD. The median pre-CK BCVA was 2.6 logMAR (range: 0.1–2.9 logMAR), with a mean pre-CK VF MD of −17.47 ± 9.41 dB (range: −31.57 to −5.49 dB). Pre-CK BCVA was 20/200 or worse in 13 eyes (59.1%, 13/22), 20/20 to better than 20/60 in 5 eyes (22.7%, 5/22), and 20/60 to better than 20/200 in 4 eyes (18.2%, 4/22) (Figure 2).

The median follow-up duration for visual function was 36 months (range: 26–64 months). The median BCVA after CK was 0.6 logMAR (range: 0–2.9 logMAR), with a mean VF MD of −11.46 ± 8.92 dB (range: −23.96 to −0.08 dB). Nine eyes (40.9%) achieved VA of 20/20 to >20/60 after CK, 3 eyes (13.6%) achieved 20/60 to >20/200, and 10 eyes (45.5%) had VA of 20/200 or worse. Table 2 summarizes the long-term visual function outcomes before and after CK.

Among the 22 affected eyes undergoing visual acuity assessment, 7 eyes (31.8%) maintained stable BCVA, 13 eyes (59.1%) showed improved BCVA, and the remaining 2 eyes (9.1%) experienced decreased BCVA post-CK. Post-CK BCVA was significantly improved compared with pre-CK BCVA (*Z* = −2.990, *p* = 0.003). We defined visual stability and improvement as treatment success, resulting in 20 eyes achieving visual treatment success. Among the 12 eyes with available VF MD data, 11 eyes showed marked improvement in VF MD, while only 1 eye demonstrated worsening VF MD. Post-CK VF MD significantly differed from pre-CK VF MD (*p* = 0.002). Table 3 summarizes the BCVA, VF MD, and overall visual function outcomes after CK.

Baseline BCVA ≤ 20/200 was observed in 13 eyes (59.1%), of which 6 (46.2%) improved, 5 remained stable (1 HM, 1 LP, and 3 NLP), and 2 declined from LP to NLP. Conversely, among 9 eyes (40.9%) with baseline BCVA > 20/200, 7 (77.8%) achieved improvement and 2 were stable. Notably, even among 5 eyes with extreme baseline impairment (CF or below), the potential for recovery was observed.

The Spearman correlation analysis of baseline clinical factors (Table 4) identified two factors significantly associated with visual outcomes. Pre-CK BCVA showed a significant positive correlation with ∆VA (*r* = 0.457, *p* = 0.032), indicating that patients with poorer baseline vision achieved less visual recovery. Furthermore, a longer time from onset to CK was significantly associated with worse visual outcomes (*r* = 0.450, *p* = 0.036). In contrast, age and tumor volume did not demonstrate a significant impact on visual changes (*p* > 0.05).

### 3.3. Tumor Control Rate

At our center, MRI follow-up is conducted every 3 or 6 months after CK completion in patients without complications. Tumor volume assessment was performed for 14 affected eyes across 13 patients who completed MRI follow-up. The median follow-up duration for these 14 eyes was 31.5 months (range: 26–64 months); the tumor control rate was 100%.

### 3.4. Radiotherapy Complications

During treatment and follow-up at our center, none of the 20 patients experienced ocular adverse events such as radiation optic neuropathy, retinopathy, vitreous hemorrhage, cataracts, dry eye, or iridocyclitis, nor systemic adverse events including vomiting, dizziness, or headache during or after CK therapy.

## 4. Discussion

In this single-center study, we systematically evaluated the long-term visual function outcomes and safety of CK hypofractionated radiosurgery (hypo-RS) in patients with ONSM. The study, which included 20 patients (22 affected eyes), demonstrated that CK treatment achieved favorable efficacy in preserving and improving visual function, with no significant radiation-related toxicities observed.

Previous studies have shown that ONSM predominantly affects middle-aged women [13,14]. The present cohort similarly consisted primarily of women (15/20, 75.0%), with a mean age at onset of 40.2 ± 15.6 years, consistent with prior reports. Notably, patients in this study exhibited significant visual impairment prior to treatment, with a median baseline BCVA of 2.6 logMAR. Nevertheless, CK therapy still resulted in visual improvement in a subset of patients with poor baseline vision, although this aspect has been less emphasized in previous studies [15].

In this study, six eyes with baseline BCVA ≤ 20/200 still experienced an improvement in visual acuity following treatment. These cases typically involved a shorter interval between disease onset and intervention, suggesting that visual function can still be improved through early CK, even in eyes with severely impaired baseline vision. This indicates that visual dysfunction associated with ONSM is primarily driven, at certain stages, by reversible conduction disturbances caused by tumor compression of the optic nerve and its blood supply, which disrupts axoplasmic transport. It is not driven by irreversible axonal degeneration occurring in the early stages [16]. Previous studies have also demonstrated that visual function can improve rapidly after radiotherapy, even when tumor volume reduction is not significant. This underscores the critical importance of promptly relieving compressive damage for visual recovery [17].

The primary finding of this study was a high rate of preserved and improved visual function. Following CK therapy, BCVA remained stable or improved in 90.9% of affected eyes, and VF MD improved in 91.7% of affected eyes. Median BCVA improved from 2.6 logMAR (range: 0.1–2.9 logMAR) to 0.6 logMAR (range: 0–2.9 logMAR), while VF MD improved from −17.47 ± 9.41 dB to −11.46 ± 8.92 dB. Both differences were statistically significant. Among the 20 patients (22 eyes), only 2 eyes showed further deterioration in BCVA, and both had only LP prior to treatment. In the 5 eyes with pre-CK BCVA ≤ 20/200 and stable VA after treatment, 1 eye had HM, 1 had LP, and 3 had NLP before CK. These findings in both groups suggest that the optic nerve damage may have approached an irreversible stage. In such cases, CK radiotherapy is unlikely to improve visual function.

Importantly, 59.1% of affected eyes demonstrated clear visual improvement following treatment, a rate higher than that observed in previous studies using other radiotherapy modalities [13,18]. Among patients with imaging follow-up, this study observed a 100% tumor control rate without acute or late radiotoxicity reactions such as radiation optic neuropathy, retinopathy, or dry eye syndrome. This favorable safety profile may be closely related to the low-fractionation radiosurgery regimen employed. These findings further support the safety and efficacy of CK as a vision-preserving treatment for ONSM and provide a valuable reference for Asian populations, for which relevant reports remain relatively limited.

In recent years, treatment strategies for ONSM have gradually shifted from observation or microsurgery (the latter often associated with a higher risk of blindness) toward a comprehensive management model centered on radiotherapy [19]. The approximately 91% visual acuity success rate in this study is comparable to recent meta-analysis findings on fractionated stereotactic radiotherapy (FSRT) and intensity-modulated radiotherapy (IMRT) (typically reported as 80–100%) [4,20,21]. However, the visual improvement rate in this study (59.1%) was relatively higher. In contrast, previous reports on stereotactic radiosurgery (SRS, such as gamma knife) indicate that vision improvement rates typically range between 30% and 40% [22,23].

Furthermore, the findings of this study indicate that baseline BCVA and the interval from symptom onset to CK were significantly associated with visual outcomes, consistent with previous studies [15,24,25]. Patients with better baseline vision were more likely to experience improvement, whereas those presenting with severe visual impairment (e.g., LP or NLP) showed limited recovery potential, likely reflecting optic nerve damage and axonal degeneration secondary to prolonged compression or ischemia. In addition, longer symptom duration was associated with poorer visual prognosis, highlighting the importance of early intervention [15]. In contrast, age and tumor volume were not significantly correlated with visual changes, suggesting that visual outcomes may depend more on the location and duration of optic nerve involvement than on tumor size alone. However, this hypothesis requires further research to confirm. Overall, baseline visual function and disease duration may serve as practical indicators for prognostic assessment and treatment timing in ONSM.

Compared with single-fraction SRS, a multi-fraction treatment regimen may facilitate the repair of sublethal damage in normal neural tissue during inter-fraction intervals while maintaining an effective therapeutic dose to the tumor [26]. However, in ONSM, the involved optic nerve lies within or immediately adjacent to the treatment target because the tumor arises from the optic nerve sheath. Thus, although stereotactic irradiation can reduce exposure to surrounding tissues, the involved optic nerve itself remains within the treatment field. Its main dosimetric advantage is therefore the reduction in unnecessary radiation exposure to surrounding ocular and orbital structures. Previous studies have suggested that the risk of radiation-induced optic neuropathy increases when the optic nerve receives a high single-fraction dose, particularly above approximately 10–12 Gy [27,28]. Fractionating the total dose over five sessions may improve the tolerance of normal neural tissue by allowing sublethal damage repair between fractions. In the present cohort, no radiation-induced optic neuropathy, retinopathy, or other serious ocular toxicities were observed during follow-up; however, these findings should be interpreted cautiously given the small sample size and limited duration of follow-up. Therefore, the five-fraction hypo-RS regimen may provide a practical compromise between the convenience of single-fraction SRS and the prolonged treatment course of conventional FSRT (25–30 fractions).

The primary limitations of this study include its retrospective, single-center design and relatively small sample size (*n* = 20), which largely reflects the rarity of ONSM and may introduce selection bias. In addition, paired reliable visual field data were available for only 12 of the 22 affected eyes, and long-term MRI follow-up was not available for all patients. These limitations reduce the precision and generalizability of the estimates. Larger multicenter studies with standardized ophthalmic and imaging follow-up are warranted.

## 5. Conclusions

In conclusion, CK hypofractionated radiosurgery was associated with preservation or improvement of visual function in most treated eyes, while tumor control was achieved in all eyes with available MRI follow-up. No serious treatment-related adverse events were observed during follow-up. These findings suggest that CK may represent a treatment option for patients with ONSM, particularly those with residual visual function.

## Figures and Tables

**Table 1:** Demographic Data and Clinical Characteristics.

Variables	Values
Patients, *n*	20
Affected eyes, *n*	22
Sex, *n* (%)	
Female	15 (75.0%)
Male	5 (25.0%)
Age at symptom onset, years	40.2 ± 15.6
Interval from symptom onset to presentation of affected eyes, months	13.5 (2–197)
Interval from symptom onset to initiation of CK of affected eyes, months	15 (2–198)
Laterality of patients, *n* (%)	
Unilateral	18 (90%)
Bilateral	2 (10%)
Side of affected eyes, *n* (%)	
Right	12 (54.5%)
Left	10 (45.5%)
Symptoms of affected eyes, *n* (%)	
Decreased visual acuity	20 (90.9%)
Visual field impairment	13 (59.1%)
Signs of affected eyes, *n* (%)	
Optic disc swelling	15 (68.2%)
Optic disc pallor	6 (27.3%)
Normal optic disc	1 (4.5%)
Proptosis	1 (4.5%)

CK, CyberKnife; SD, standard deviation. Data are presented as mean ± SD, median (range), or *n* (%), as appropriate. Symptoms and signs were analyzed based on affected eyes.

**Table 2:** Long-term visual function outcomes before and after CK.

Visual Function	Before CyberKnife	After CyberKnife	*p*-Value
BCVA, logMAR, median (range)	2.6 (0.1–2.9)	0.6 (0–2.9)	0.003 *
BCVA category, *n* (%)			
20/60 < BCVA ≤ 20/20	5 (22.7%)	9 (40.9%)	
20/200 < BCVA ≤ 20/60	4 (18.2%)	3 (13.6%)	
BCVA ≤ 20/200	13 (59.1%)	10 (45.5%)	
VF MD, dB, mean ± SD ^†^	−17.47 ± 9.41	−11.46 ± 8.92	0.002 *

CK, CyberKnife; BCVA, best-corrected visual acuity; logMAR, logarithm of the minimum angle of resolution; VF MD, visual field mean deviation; SD, standard deviation. Data are presented as median (range), mean ± SD, or *n* (%). ^†^ VF MD analysis included 12 eyes with reliable paired visual field examinations. * *p* < 0.05.

**Table 3:** BCVA, VF MD, and Overall Visual Function Outcomes After CK.

Characteristic	Number	Percentage
BCVA, *n* of affected eyes	22	
Stable	7	31.8%
Improved	13	59.1%
Worsened	2	9.1%
Treatment success	20	90.9%
VF MD, *n* of affected eyes	12	
Improved	11	91.7%
Worsened	1	8.3%
Treatment success	11	91.7%
Overall visual function, *n* of affected eyes	12	
Improved	11	91.7%
Worsened	1	8.3%
Treatment success	11	91.7%

CK, CyberKnife; BCVA, best-corrected visual acuity; VF MD, visual field mean deviation. Treatment success was defined as stable or improved visual function after CK treatment.

**Table 4:** Spearman Correlation Analysis of Factors Associated with VA Change.

Variable Pairs	*r*	*p*-Value
Age, years	0.044	0.845
Pre-CK BCVA, logMAR	0.457	0.032 *
Tumor volume, mm^3^	−0.165	0.464
Interval from symptom onset to CK, months	0.450	0.036 *

Pre-CK, before CyberKnife; BCVA, best-corrected visual acuity; VA, visual acuity; *r*, Spearman’s rank correlation coefficient. * *p* < 0.05.

## Data Availability

The data presented in this study are available on request from the corresponding author due to data management requirements.

## Abbreviations

The following abbreviations are used in this manuscript:

ONSM	Optic nerve sheath meningiomas
STR	Stereotactic radiotherapy
IMRT	Intensity-modulated radiation therapy
CK	CyberKnife
VA	Visual acuity
VF	Visual field
BCVA	Best-corrected visual acuity
CF	Counting fingers
HM	Hand motion
LP	Light perception
NLP	No light perception
Hypo-RS	Hypofractionated radiosurgery
FSRT	Fractionated stereotactic radiotherapy
